# Quality of sleep among trainee doctors at the Charles Nicolle Hospital after vaccination against COVID19

**DOI:** 10.1192/j.eurpsy.2024.1077

**Published:** 2024-08-27

**Authors:** Z. Athimni, G. Bahri, M. Mersni, I. Youssef, D. Brahim, H. Ben Said, N. Mechergui, N. Ladhari, K. Imene

**Affiliations:** Department of Occupational Pathology and Fitness for Work - Charles Nicolle Hospital, Tunis, Tunisia

## Abstract

**Introduction:**

Sleep quality depends on several factors such as smoking, physical activity, diet, and certain pathologies, namely obstructive sleep apnoea syndrome. Indeed, following their vaccination against COVID19, several medical trainees complained about a deterioration of their sleep quality.

**Objectives:**

To evaluate the quality of sleep of medical trainees who work at Charles Nicolle Hospital and who were vaccinated against SARS-COV2.

**Methods:**

We conducted a descriptive cross-sectional study among medical trainees at Charles Nicolle Hospital who were vaccinated against COVID-19 during the period from March 2020 to August 2022. Sleep quality was evaluated by the Pittsburgh Sleep Quality Index (PSQI) questionnaire. Trainees were contacted during the period August 2022 to September 2022.

**Results:**

Sixty-nine medical trainees, vaccinated against Covid19 joined our study. Forty-nine of them had a significant sleep disturbance: Pittsburgh Sleep Quality Index (PSQI) greater than five. The average age was 29.39±3.04 years with a female majority (73.5%). No psychiatric history was found. The most affected category of trainees were residents (71.4%). Forty-three of them were inoculated with the messenger RNA vaccine and 4 with inactivated vaccine. Twenty-one patients vaccinated with the messenger RNA vaccine received two doses, seventeen received three doses and only one received a single dose. Sleep latency was high in 20,4% of cases. A sleep duration of less than five hours per night was found in 18,4% of the cases. Six participants reported using a sleep aid three to four times a week.

**Conclusions:**

Our study revealed a significant sleep disturbance in medical trainees at Charles Nicolle Hospital. This could be due to the SARS-COV2 vaccination but can also be explained by the night shifts and the stress to which they are exposed, especially during this pandemic period.

**Disclosure of Interest:**

None Declared

